# STAT1-Deficient HPV E6/E7-Associated Cancers Maintain Host Immunocompetency against Therapeutic Intervention

**DOI:** 10.3390/vaccines12040430

**Published:** 2024-04-17

**Authors:** Ling Lim, Ming-Hung Hu, Darrell Fan, Hsin-Fang Tu, Ya-Chea Tsai, Michelle Cheng, Suyang Wang, Chih-Long Chang, Tzyy-Choou Wu, Chien-Fu Hung

**Affiliations:** 1 Department of Pathology, Johns Hopkins University School of Medicine, Baltimore, MD 21231, USAwutc@jhmi.edu (T.-C.W.); 2 Department of Obstetrics and Gynecology, MacKay Memorial Hospital, Taipei 104217, Taiwan; clchang@mmc.edu.tw; 3 Graduate Institute of Clinical Medicine, College of Medicine, National Taiwan University, Taipei 100, Taiwan; 4 Division of Hematology and Oncology, Department of Internal Medicine, Wan Fang Hospital, Taipei Medical University, Taipei 11031, Taiwan; 5 Cancer Center, Wan Fang Hospital, Taipei Medical University, Taipei 11031, Taiwan; 6 Department of Oncology, Johns Hopkins University School of Medicine, Baltimore, MD 21231, USA; 7 Department of Obstetrics and Gynecology, Johns Hopkins University School of Medicine, Baltimore, MD 21231, USA; 8 Molecular Microbiology and Immunology, Johns Hopkins University School of Medicine, Baltimore, MD 21231, USA

**Keywords:** STAT1, human papillomavirus, E6/E7, T cells, immunity

## Abstract

Human papillomavirus (HPV) remains a global health concern because it contributes to the initiation of various HPV-associated cancers such as anal, cervical, oropharyngeal, penile, vaginal, and vulvar cancer. In HPV-associated cancers, oncogenesis begins with an HPV infection, which is linked to the activation of the Janus protein tyrosine kinase (JAK)/STAT signaling pathway. Various STAT signaling pathways, such as STAT3 activation, have been well documented for their tumorigenic role, yet the role of STAT1 in tumor formation remains unclear. In the current study, STAT1^−/−^ mice were used to investigate the role of STAT1 in the tumorigenesis of a spontaneous HPV E6/E7-expressing oral tumor model. Subsequently, our candidate HPV DNA vaccine CRT/E7 was administered to determine whether the STAT1^−/−^ host preserves a therapeutic-responsive tumor microenvironment. The results indicated that STAT1^−/−^ induces robust tumorigenesis, yet a controlled tumor response was attained upon CRT/E7 vaccination. Characterizing this treatment effect, immunological analysis found a higher percentage of circulating CD4+ and CD8+ T cells and tumor-specific cytotoxic T cells. In addition, a reduction in exhaustive lymphocyte activity was observed. Further analysis of a whole-cell tumor challenge affirmed these findings, as spontaneous tumor growth was more rapid in STAT1^−/−^ mice. In conclusion, STAT1 deletion accelerates tumorigenesis, but STAT1^−/−^ mice maintains immunocompetency in CRT/E7 treatments.

## 1. Introduction

Globally, human papillomavirus (HPV) remains the most prevalent sexually transmitted viral infection, with a rising incidence rate each year [1,2]. This global health burden is attributed to over 200 HPV types, categorized by oncogenic potential from low to high risk [3,4]. While most low-risk HPV infections regress or remain subclinical, a subset of high-risk HPVs lead to cancerous growth within 10–30 years [5]. To this end, HPV has been established as the primary etiological factor for various cancers, including anal, cervical, oropharyngeal, penile, vaginal, and vulvar cancer [6,7,8]. Among these HPV types, high-risk HPV (HR-HPV) types 16 and 18 account for approximately 70–90% of all oropharyngeal and cervical cancers [6,9,10,11]. Furthermore, the viral oncoproteins HPV E6 and E7 (HPV-E6/E7) play a necessary and critical role in the initiation and maintenance of HPV-associated cancers [12].

Following HPV infection, pattern-recognition receptors identify the viral components and induce interferon synthesis, triggering the Janus protein tyrosine kinase (JAK)/STAT signaling pathway [13]. The signal transducer and activator of transcription (STAT) protein family, a group of intracellular transcription factors, orchestrates a wide array of key biological processes, spanning from cell differentiation to immune system regulation [14]. Numerous studies have well documented the pro-tumorigenic effects of STAT3 activation, correlating this expression with the development of more severe lesions and a poorer clinical prognosis in HPV16-associated cancer cases [15,16,17,18,19]. However, the precise role of STAT1 in tumorigenesis remains unclear, presenting an area for further investigation.

STAT1 is a cytoplasmic transcription factor comprising six distinct domains, and it activates in response to both type I and II interferon (IFN) stimulation. Activation ensues through the tyrosine phosphorylation-mediated dimerization of the SH2 domain, mediated by JAKs [20]. Activated STAT1 can form multimers with other STAT proteins and translocate into the nucleus, where it exerts cis-regulatory control over downstream DNA sequences [21,22,23]. As such, STAT1 expression influences a myriad of physiological processes such as apoptosis, inflammatory responses, and cell cycle modulation [14,20].

STAT1 deficiency (STAT1^−/−^) has emerged as a feature in the pathobiology of many cancers, including breast cancer, colorectal cancer, esophageal cancer, and lung cancer [24,25,26,27]. While the loss of STAT1 is a common occurrence in tumorigenesis, there are different perspectives regarding its role in cancer immunology. On the one hand, certain studies demonstrated that STAT1 activation exhibits antitumor properties by enhancing IFN-α and -γ-dependent intracellular responses and downregulating the PD-1/PD-L1 axis in cancers such as non-small-cell lung cancer (NSCLC), colorectal cancer, and head and neck squamous cell carcinoma (HNSCC) [28,29,30,31,32,33]. Conversely, other studies determined that STAT1 promotes tumorigenesis, fostering chemoresistance and radioresistance in cancers like renal cell carcinoma, breast cancer, and myeloma [34,35,36]. In HPV-associated cervical cancers, patients with normal or upregulated STAT1 expression often present with higher-grade lesions, yet they exhibit longer overall survival compared to those patients with STAT1^−/−^ [37]. Similarly, in HPV-associated HNSCC, STAT1^−/−^ is associated with downregulation of antigen processing, heightened immunosuppression, and a higher incidence of metastasis [28,38,39]. Therefore, it remains imperative to continue scrutinizing the effects of STAT1 on the progression of HPV-associated cancers.

In this study, we investigated the effect of STAT1^−/−^ on the tumorigenesis of a spontaneous HPV E6/E7-expressing oral tumor model. Subsequently, we administered our candidate HPV DNA vaccine CRT/E7 to determine whether a therapeutic-responsive tumor microenvironment was still preserved. Our hypothesis posits that STAT1^−/−^ induces robust tumorigenesis, yet the host immune system does not become entirely treatment-resistant. This observation is illustrated by a controlled tumor response upon CRT/E7 vaccination, which affirms that the host immune system remains responsive and intact. This observed treatment effect is characterized by a higher percentage of activated tumor-infiltrating CD4+ and CD8+ T cells, coupled with a reduction in exhaustive lymphocyte activity.

## 2. Materials and Methods

### 2.1. Mice and Animal Care

Six- to eight-week-old female C57BL/6NTac mice were purchased from Taconic Biosciences (Rensselaer, NY, USA). STAT1^−/−^ mice were purchased from Jackson Laboratories (Bar Harbor, ME, USA) and bred in our in-house animal facility. All mice were maintained under specific pathogen-free conditions at the Johns Hopkins University School of Medicine Animal Facility (Baltimore, MD, USA). All animal procedures were conducted per recommendations for the proper use and care of laboratory animals.

### 2.2. Plasmid DNA Construction and Preparation

The generation of Pkt2-Luc-T2a-E7-T2a-E6 plasmid (E6/E7 oncogenes and luciferase), pT/Caggs-NrasV12 plasmid (Nras^G12v^), and pCMV(CAT)T7-SB100 plasmid (SB100) has been described previously [40,41,42]. The generation of pcDNA-3-CRT/E7 (CRT/E7) has also been described previously [43].

### 2.3. Generation of Spontaneous Oral Tumor Model

Six- to eight-week-old female STAT1^−/−^ mice were utilized for the development of the oral tumor model. The mice were first anesthetized intramuscularly with an 80 μL solution composed of 16.7% Ketaset (100 mg/mL), 16.7% AnaSed (20 mg/mL), and 66.6% PBS. Then, 10 μg of plasmids encoding HPV16-E6/E7, luciferase, mutant NRas^G12V^, and SB100 was injected into the buccal mucosa of each mouse (30 μL/injection). This was immediately followed by electroporation at the injection site using BTX830 (BTX Harvard Apparatus, Holliston, MA, USA). An electrode tweezer was used to deliver the following electroporation parameter: 72 V for 20 ms with intervals of 200 ms for 8 pulses. Tumor growth was closely followed via luminescent imaging.

### 2.4. Bioluminescent Imaging

Tumor growth was monitored weekly for a total of four weeks after initial injection with E6/E7 oncogenes. The mice received intraperitoneal injection (IP) with D-luciferin (GoldBio, Olivette, MO, USA) substrate before being anesthetized in an isoflurane chamber. After ten minutes, bioluminescent imaging for luciferase expression was performed in the IVIS Spectrum in vivo imaging system series 2000 (PerkinElmer, Shelton, CT, USA) using the Living Image acquisition and analysis software (Xenogen, Tucson, AZ, USA). The tumor region was quantified as photon flux of the luciferase expression using the Living Image 2.50 software (Xenogen).

### 2.5. DNA Vaccination

Before HPVE6/E7 oncogene injection, the mice were vaccinated subcutaneously, followed by electroporation, in the shaved left rear flank twice at 1-week intervals with 10 µg of the CRT/E7 DNA vaccine. Tumor growth was monitored once a week via bioluminescent imaging. Tumor-bearing mice reached the experimental endpoint when body weight loss was experienced or when the total photon flux exceeded 1 × 10^9^ p/sec/cm^2^/sr.

### 2.6. Flow Cytometry Analysis

For the analysis of peripheral blood mononuclear cells (PBMCs), blood samples were obtained from the submandibular vein of mice and collected into 10% EDTA-coated tubes. Red blood cells (RBCs) were lysed twice using an RBC lysis buffer (Cell Signaling Technology, Danvers, MA, USA).

For the analysis of spleen and tumor cells, tumors and spleens were surgically excised from the mice and placed in RPMI-1640 medium. Spleen samples were minced into smaller pieces. Tumor samples underwent incubation in a tissue digestion buffer containing DNase I (100 U/mL), collagenase I (0.05 mg/mL), and collagenase IV (0.05 mg/mL), followed by dissociation using the gentleMACS^TM^ Tissue Dissociator (Miltenyi Biotec, Gaithersburg, MD, USA). Digested tumor samples were filtered through a 70 μM nylon cell strainer. After centrifugation and extensive washing using 0.5% BSA/PBS (FACs buffer), tumor-infiltrating mononuclear cells were separated from tumor immune cells through establishing a Ficoll gradient using Ficoll-Paque plus (GE Healthcare Life Sciences, Marlborough, MA, USA).

All samples were plated at approximately equal amounts, and Fc blocking was applied before antibody staining. Zombie Aqua live/dead staining (BioLegend, San Diego, CA, USA) facilitated live and dead cell discrimination. Antibody staining included PerCP-conjugated anti-mouse CD3, BV780-conjugated anti-mouse CD4, APC-A750-conjugated anti-mouse CD8a, FITC-conjugated anti-mouse PD1, APC-conjugated anti-mouse Foxp3, APC-A750-conjugated anti-mouse CD25, BV780-conjugated anti-mouse Ly6G, and BV421-conjugated anti-mouse Ly6C. Staining for E7-specific immune populations involved incubating samples with a PE-conjugated HPV16 E7 (aa 49 to 57) tetramer (1 mg/mL).

Flow cytometry data for all samples were collected using a 13-color Beckman Coulter CytoFLEX S with the CytExpert software (version 2.3.1.22). Single-color bead controls for fluorochromes used in each experiment were measured. The compensation matrix was calculated based on unstained cells, unstained beads, and beads of each individual fluorochrome. A compensation matrix was then applied to experimental samples using the CellQuest software. Data analysis was completed using the FlowJo 10.4 software (FlowJo LLC, Ashland, OR, USA).

### 2.7. Cell Culture

TC-1 cells were maintained in DMEM media supplemented with 10% FBS, 1% L-glutamine, 100 mg/mL streptomycin, 100 U/mL penicillin, 2 mM of sodium pyruvate, and 2 mM of non-essential amino acid.

### 2.8. Tumor Challenge Experiment

The CRT/E7 vaccination group was treated as described before; then, all mice were inoculated with 2 × 10^4^ TC-1 cells (50 μL PBS) on the right flank subcutaneously. Tumor growth was measured 2–3 times a week with a digital caliper. Tumor volume was calculated using the following formula: 0.5 × length × width × width. Tumor-bearing mice reached the experimental endpoint when body weight loss was experienced or when the tumor volume exceeded 1500 mm^3^, or 20 mm in length, in accordance to our animal protocol.

### 2.9. Statistical Analysis

All data are presented as mean ± standard error (S.E.M.). Statistical significance was determined using one-way ANOVA with Tukey–Kramer multiple comparison or Student’s *t*-test on GraphPad Prism V.10 software (La Jolla, CA, USA). All *p*-values ≤ 0.05 were considered significant (* = *p* < 0.05; ** = *p* < 0.01; *** = *p* < 0.001; **** *p* < 0.0001).

## 3. Results

### 3.1. Formation of Spontaneous HPV+ Oral Tumors in STAT1^−/−^ Mice

**Figure 1:**
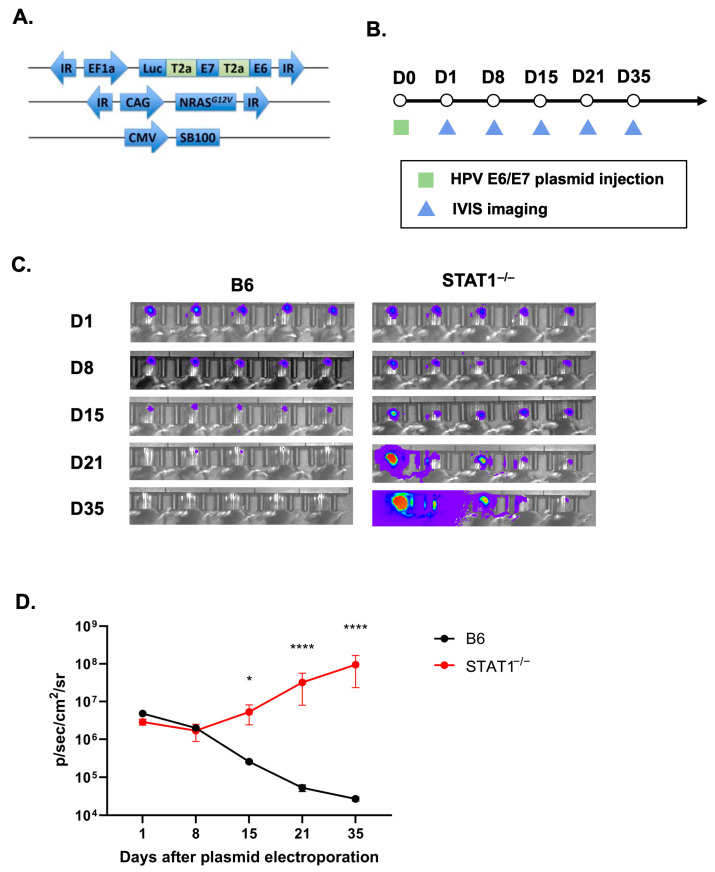
STAT1^−/−^ mice promote tumorigenesis of HPV oral tumor model. (**A**) Schematic diagram of the three DNA plasmids encoding HPV16 E6/E7 with luciferase, Ras, and SB100 DNA used for tumor model. (**B**) Schematic diagram of the experimental procedure; 4–6-weeks-old C57BL/6NTac mice and STAT1^−/−^ mice were injected with DNA plasmids encoding HPV16 E6/E7 with luciferase, Ras, and SB100 (10 μg/plasmid, 30 μL/injection per mouse) into the buccal mucosa, followed by electroporation. Tumor growth in mice was monitored via IVIS imaging weekly (*n* = 5). (**C**) Bioluminescence imaging of tumor-bearing mice using IVIS Spectrum after IP injection of luciferin solution (*n* = 5). (**D**) Line graph summarizing the bioluminescence imaging of the tumor growth over time (*n* = 5). All data are presented as the mean ± SEM (* *p* < 0.05; **** *p* < 0.0001).

To investigate the effects of STAT1 on tumorigenesis, all C57BL/6NTac (control) mice and STAT1^−/−^ mice were given the plasmid combination containing E6/E7 oncogenes conjugated with luciferase, NRAS^G12V^, and SB100 in the buccal mucosa (Figure 1A). Bioluminescent imaging was conducted on designated days (Figure 1B) to discern differences in tumor growth between the two groups. The localized luciferase intensity began diminishing after day 8 in the control group, demonstrating a failure to establish the tumor. In contrast, STAT1^−/−^ mice exhibited significantly greater and sustained spontaneous tumor growth compared to the control group (Figure 1D). These observations indicated that STAT1 plays a role in controlling the growth of HPV E6/E7-expressing oral tumors in vivo.

### 3.2. Vaccination with CRT/E7 DNA Induces Tumor Control in STAT1^−/−^ Mice

**Figure 2:**
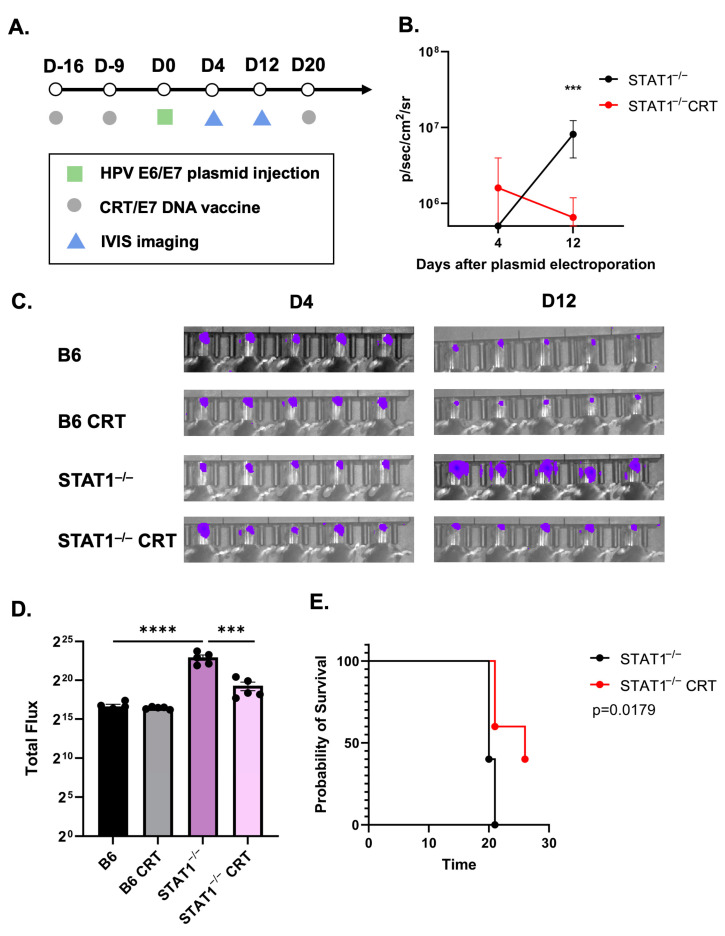
CRT/E7 vaccination showcased antitumor effect against spontaneous HPV tumors in STAT1^−/−^ mice. Mice were administrated with HPV16 E6/E7 plasmid (10 ug) followed by electroporation as described. (**A**) Schematic of therapeutic HPV E6/E7 vaccination with CRT/E7 experiment design. (**B**) Line graph illustrating the total luciferase intensity of the tumor after CRT/E7 vaccination treatment (*n* = 5). (**C**) Individual bioluminescence imaging among each group after vaccine administration (*n* = 5). (**D**) Bioluminescence intensity comparison in each group at the end of treatment (*n* = 5). (**E**) Survival curve is presented in percentages (*n* = 5). All data are presented as the mean ± SEM (*** *p* < 0.001; **** *p* < 0.0001).

Next, it was crucial to determine whether the HPV E6/E7-expressing oral tumor could be controlled in STAT1^−/−^ mice. The HPV DNA vaccine CRT/E7 was used to assess how the spontaneous tumor would be affected. One group of control mice and STAT1^−/−^ mice were vaccinated with two doses of CRT/E7 before being injected with E6/E7 oncogenes in the buccal mucosa (Figure 2A). Bioluminescent imaging conducted on days 4 and 12 revealed a significant reduction in total luciferase intensity in vaccinated STAT1^−/−^ mice compared to unvaccinated STAT1^−/−^ mice (Figure 2B,C). In addition, vaccinated STAT1^−/−^ mice demonstrated significantly less total luciferase intensity than unvaccinated STAT1^−/−^ mice, suggesting a better controlled tumor response in vaccinated STAT1^−/−^ mice (Figure 2D). When comparing the unvaccinated groups, unvaccinated STAT1^−/−^ mice showed significantly greater total luciferase intensity than unvaccinated control mice, which is consistent with the results from Figure 1 (Figure 2D). The survival curve further revealed that vaccinated STAT1^−/−^ mice exhibited significantly longer overall survival compared to unvaccinated STAT1^−/−^ mice. All unvaccinated STAT1^−/−^ mice died within 21 days of tumor formation, whereas two vaccinated STAT1^−/−^ mice survived until the experimental endpoint (Figure 2E). Collectively, these findings showed that the endogenous immune system of STAT1^−/−^ mice remained responsive to CRT/E7 vaccination.

### 3.3. CRT/E7 Vaccination Contributes to the Enhancement of Effector T-Cell Function in STAT1^−/−^ Mice

**Figure 3:**
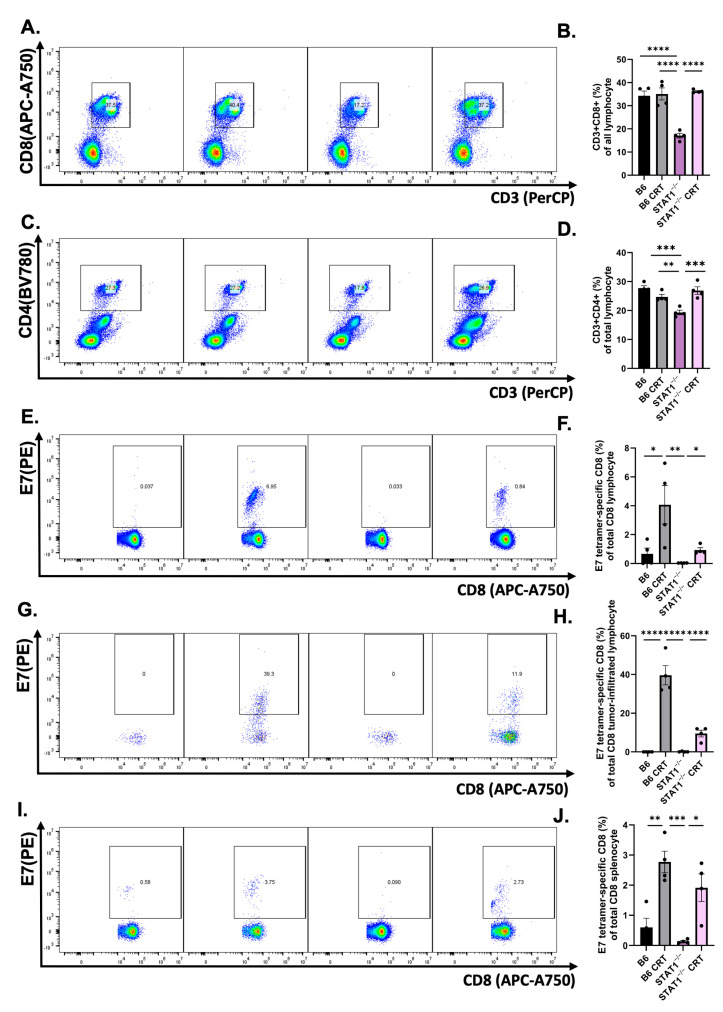
CRT/E7 vaccine-induced effector T cells lead to tumor control in STAT1^−/−^ mice. Therapeutic CRT/E7 DNA vaccine administration displays greater effector T-cell population. TC-1-bearing mice were administered with CRT/E7 vaccine for a total of three cycles in 7-day intervals. Electroporation followed immediately after DNA injection. Mice PBMCs were collected on day 16. (**A**,**B**) Representative flow gating and quantification of CD8+ T cells (*n* = 5) and (**C**,**D**) CD4+ T-cell population for the indicated treatment groups (*n* = 5). (**E**,**F**) Representative flow gating and quantification of E7-tetramer-specific CD8+ T cells (*n* = 5). Mice were euthanized on day 20 and TILs were isolated. (**G**,**H**) Representative flow gating and quantification of E7-tetramer-specific CD8+ TILs (*n* = 5). (**I**,**J**) Representative flow gating and quantification of E7-tetramer-specific CD8+ splenocytes (*n* = 5). All data are presented as the mean ± SEM (* *p* < 0.05; ** *p* < 0.01; *** *p* < 0.001; **** *p* < 0.0001).

Given the controlled tumor response observed in STAT1^−/−^ mice, it is critical to examine the immunological response elicited by CRT/E7. This assesses the immunocompetency of STAT1^−/−^ mice. PBMCs were collected from tumor-bearing mice as described in the protocol, and T-cell populations were analyzed with flow cytometry. The vaccinated STAT1^−/−^ mice exhibited significantly elevated CD8+ (Figure 3A,B) and CD4+ T-cell (Figure 3C,D) populations compared to unvaccinated STAT1^−/−^ mice. Subpopulation analysis revealed a significant increase in E7-specific CD8+ T cells in STAT1^−/−^ mice subjected to CRT/E7 vaccination compared to those without vaccination (Figure 3E,F). Tumors and spleens were also collected from tumor-bearing mice as described in the protocol to determine whether a similar trend existed in E7-specific tumor-infiltrating lymphocytes (TILs) and splenocytes. E7-specific CD8+ TILs were observed in significantly higher percentages in vaccinated STAT1^−/−^ mice than those without vaccination (Figure 3G,H). E7-specific CD8+ TILs were also observed in significantly higher percentages in vaccinated STAT1^−/−^ mice than those without vaccination (Figure 3I,J). Altogether, these findings indicate that effector T cells can be augmented in STAT1^−/−^ mice through CRT/E7 vaccination.

### 3.4. CRT/E7 Vaccination Contributes to a Reduction in T-Cell Exhaustion in STAT1^−/−^ Mice

**Figure 4:**
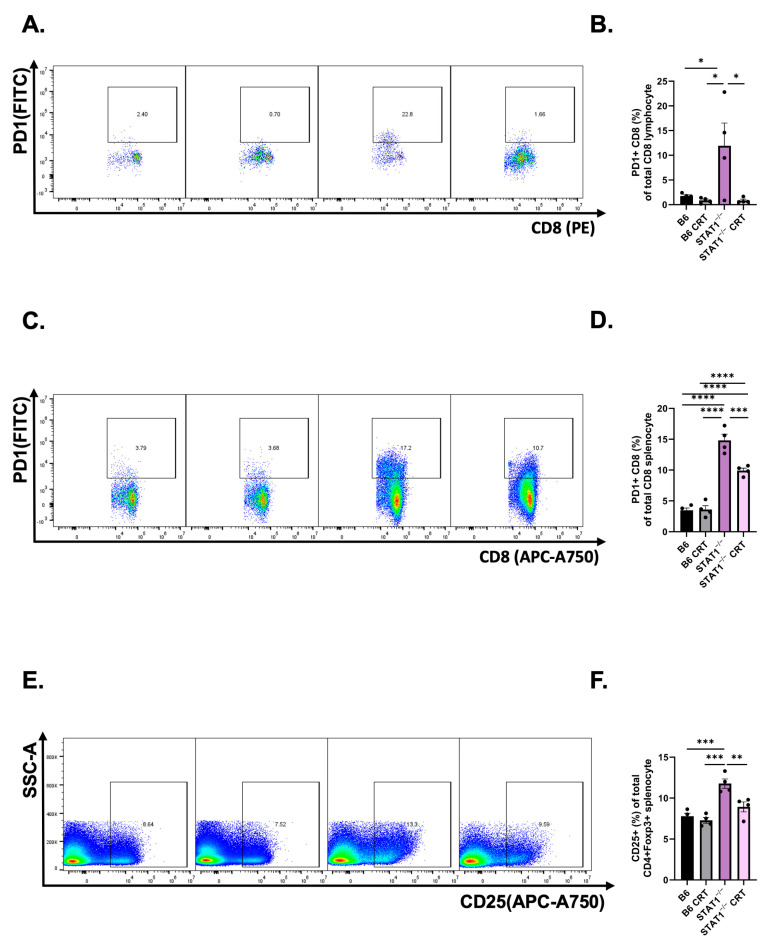
Diminished exhausted T-cell population was founded after CRT/E7 vaccination in STAT1^−/−^ mice. CRT/E7 vaccination revealed a decreased percentage of exhausted T-cell population. PBMCs were collected in TC-1-bearing mice following the described treatment protocol. (**A**,**B**) Representative flow gating and quantification of PD1-specific CD8+ T cells for the indicated treatment groups (*n* = 5). (**C**,**D**) Representative flow gating and quantification of regulatory T cells for the indicated treatment groups (*n* = 5). (**E**,**F**) Representative flow gating and quantification of PD1-specific CD8+ splenocytes for the indicated treatment groups (*n* = 5). All data are presented as the mean ± SEM (* *p* < 0.05; ** *p* < 0.01; *** *p* < 0.001; **** *p* < 0.0001).

Subsequently, we investigated alterations in T-cell exhaustion and immunosuppression. PD1-specific CD8 T cells and regulatory T cells were analyzed from mice PBMCs. Flow cytometry analysis revealed that vaccinated STAT1^−/−^ mice exhibited significantly fewer PD1-specific CD8 T cells compared to unvaccinated STAT1^−/−^ mice (Figure 4A,B). As predicted, flow cytometry analysis revealed that the control vaccinated and unvaccinated mice exhibited significantly fewer TIM3-CD4 T cells compared to the vaccinated and unvaccinated STAT1^−/−^ mice (Figure S1). Isolated splenocytes from the spleen revealed a significant reduction in PD1-specific CD8 splenocytes in vaccinated STAT1^−/−^ mice compared to unvaccinated STAT1^−/−^ mice (Figure 4C,D). Furthermore, lower proportions of regulatory T cells were presented in STAT1^−/−^ mice after vaccination (Figure 4E,F). Altogether, these analyses suggest that T-cell exhaustion is reduced in STAT1^−/−^ mice upon CRT/E7 vaccination.

### 3.5. The Host Immune Response Remains Responsive against Tumor Challenge in STAT1^−/−^ Mice

**Figure 5:**
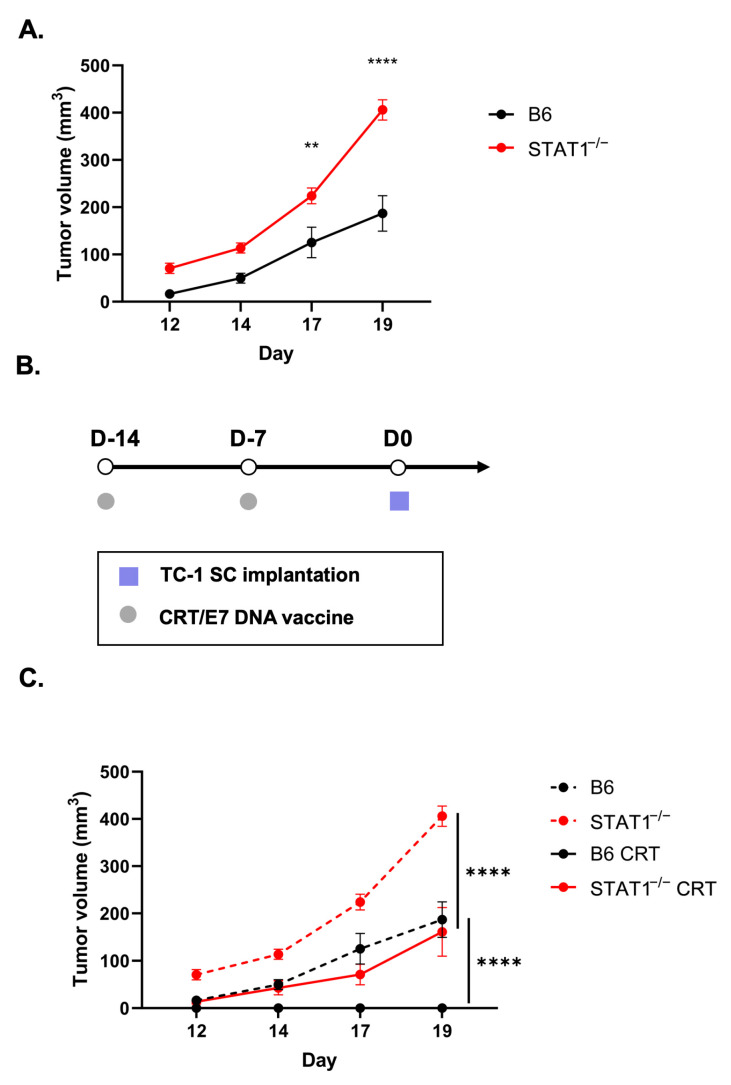
HPV DNA vaccine elicits host immune protection against tumor challenge in STAT1^−/−^ mice. (**A**) Tumor growth curve in TC-1-bearing C57BL/6NTac mice and STAT1^−/−^ mice (*n* = 5). (**B**) Schematic of prophylactic HPV E6/E7 vaccination with CRT/E7 experiment design. (**C**) Tumor growth curve following the described treatment protocol (*n* = 5). All data are presented as the mean ± SEM (** *p* < 0.01; **** *p* < 0.0001).

The above findings showcase STAT1^−/−^ mice retaining some degree of immunocompetency when vaccinated with CRT/E7. In addition, we further challenged the control and STAT1^−/−^ mice with direct tumor cell implantation to elucidate their retention of a responsive host immune system. Without CRT/E7 vaccination, TC-1-bearing STAT^−/−^ mice showed a significantly more rapid tumor growth than control mice, nearly doubling the tumor volume of the control mice by day 19 (Figure 5A). To test the host immune response against tumor challenge, mice in the vaccination groups received two doses of the CRT/E7 DNA vaccine before undergoing TC-1 tumor cell implantation (Figure 5B). The tumor growth curve demonstrated that vaccinated STAT1^−/−^ mice exhibited significantly slower tumor progression than the unvaccinated STAT1^−/−^ mice (Figure 5C). There was no tumor growth in the vaccinated control mice, showing that CRT/E7 was able to fully protect this group (Figure 5C). In essence, this finding revealed that the host immune response in STAT1^−/−^ mice is weaker than that of control mice. Nevertheless, while the vaccinated STAT1^−/−^ mice did not resist tumor growth entirely, the host immune system in STAT1^−/−^ mice remains responsive to CRT/E7 vaccination by significantly slowing down tumor progression. Once again, this underscores how deletion of STAT1 in mice allows them to stay responsive to CRT/E7 vaccination, although it does not facilitate full tumor growth protection like that in vaccinated control mice.

## 4. Discussion

In this study, we examined the role of STAT1^−/−^ in promoting the natural course of histopathological tumor progression in HPV-associated cancers. First, STAT1^−/−^ mice had greater tumor growth than control mice (Figure 1). Upon CRT-E7 administration, STAT1^−/−^ mice responded to the vaccine by demonstrating tumor control (Figure 2). Most notably, analysis of this immunological response showed that CRT-E7 treatment significantly increased the E7-specific CD8+ T cells (Figure 3) and decreased the PD-1 immune cells (Figure 4) in STAT1^−/−^ mice. Lastly, the whole-cell tumor challenge further validated that vaccinated STAT1^−/−^ mice can demonstrate tumor control because their host immune system remains intact (Figure 5).

Our initial analysis of the immunological response against CRT-E7 showed that vaccinated STAT1^−/−^ mice had significantly increased CD4+ and CD8+ T cells, and most importantly, the E7-specific CD8 T-cell population was also increased (Figure 3). Our previous studies in developing and testing CRT-E7 demonstrated its antitumor effect by significantly increasing E7-specific CD8+ T cells [43,44,45]. Therefore, the tumor control in STAT1^−/−^ mice can be attributed to the E7-specific CD8+ T-cell population.

Further analysis revealed that STAT1^−/−^ mice presented elevated expression of PD-1 immune cells compared to control mice, suggesting greater inherent immunosuppression in STAT1^−/−^ mice (Figure 4A). This observation is consistent with previous studies examining the role of STAT1^−/−^ [28,39]. Our data suggested that STAT1 is closely correlated with PD1 immune cell expression, which is supported by previous studies [46,47,48]. In addition, we demonstrated a significant increase in TIM3+ CD4+ T cells in vaccinated STAT1^−/−^ mice compared to vaccinated control mice (Figure S1). However, there were no differences in TIM3+ CD4+ T cells between unvaccinated and vaccinated STAT1^−/−^ mice, suggesting that the STAT1 pathway is more associated with PD-1 expression. Nonetheless, other exhaustion markers such as LAG3 or TIGIT could have also been used to examine T-cell exhaustion more comprehensively.

An intriguing question, then, arises regarding the responsiveness of STAT1^−/−^ hosts to therapeutic interventions, given the indispensable role of STAT1 in modulating IFN-α and -γ-dependent secretions. The immunological response is weaker in STAT1^−/−^ mice than in control mice since we observed that vaccinated control mice possessed a greater population of E7-specific CD8 lymphocytes and splenocytes compared to vaccinated STAT1^−/−^ mice (Figure 3C–E). Therefore, we further evaluated this phenomenon through a whole-cell tumor challenge to examine whether an intact and responsive host immune system is necessary for an antitumor response to be elicited in STAT1^−/−^ mice [49]. Our results confirmed that vaccinated STAT1^−/−^ mice managed to slow tumor growth, while vaccinated control mice prevented tumor growth. 

Elucidating the precise role of STAT1 has been a focal point of investigation in pathogenic and immuno-oncology studies. In pathogenic studies, the use of STAT1^−/−^ mice has facilitated extensive research on various pathogens such as Cryptococcus neoformans, lymphocytic choriomeningitis virus, Crimean–Congo hemorrhagic fever virus, and Machupo virus [50,51,52,53]. There is a consensus that upregulation of STAT1 activation confers antiviral properties, with STAT1 deficiency often increasing the susceptibility to death from viral infections [13,54]. Ultimately, these pathogenic studies established the foundation for understanding the critical role of STAT1 in immune regulation. However, there is still debate on the role of STAT1 in immuno-oncology, either as a tumor promoter or suppressor, depending on the specific cancer type. For instance, Kovacic et al. proposed STAT1 as a tumor promoter gene in hematopoietic tumors [55]. Their results showed that STAT1^−/−^ mice with leukemia exhibited low MHC type I expression to facilitate natural killer cell lysis for tumor clearance. On the other hand, STAT1^−/−^ is linked with breast cancer tumorigenesis, where the spontaneous formation of α-positive and α-negative estrogen receptors is amplified [24,56]. Hence, the different roles of STAT1 in tumorigenesis can be interpreted as either cell-autonomous or cell-nonautonomous mechanisms that predominate in tumor formation, dependent on the types of cancer involved.

The dual role of STAT1 in tumorigenesis underscores the complex relationship between STAT1 expression and therapeutic interventions. On the one hand, STAT1 activation has been linked to enhanced tumor resistance against chemotherapy, immunotherapy, and radiotherapy. For example, Palakurthi et al. found that inhibiting STAT1 expression could sensitize the tumor microenvironment to immune checkpoint blockade (ICB) treatments like anti-PD-1 in triple-negative breast cancer [57]. Most efforts to circumvent this issue involve downregulating STAT1 expression via concomitant administration of STAT1 inhibitors, such as the use of Epigallocatechin 3-gallate (EGCG) against endocrine-resistant breast cancer reported by Huang et al. [34]. On the other hand, recent studies highlighted the significance of STAT1 activation in downregulating the PD-1/PD-L1 axis. For instance, Zemek et al. reported that activating the STAT1/NK axis could sensitize the tumor microenvironment of melanoma and urothelial cancer for ICB [31]. A similar phenomenon is observed in NSCLC where STAT1^−/−^ upregulates PD-1/PD-L1 signaling, resulting in an impaired TNFα-mediated immunological response [32]. As such, most efforts to increase therapeutic efficacy involve restoring STAT1 expression. For example, Liang et al. demonstrated the use of a proteasome inhibitor, bortezomib, to restore STAT1 expression in colorectal cancer, leading to an increase in major histocompatibility complex (MHC) I expression for subsequent immunological activation [33].

Our investigation distinguishes itself from analogous studies in our alternative approach to explore the immunological response in STAT1^−/−^ mice. Instead of employing a strategy to restore STAT1 expression to enhance sensitivity to treatment interventions, we found that direct administration of our candidate DNA vaccine CRT/E7 alone was sufficient to control tumor growth in STAT1^−/−^ mice. One plausible explanation for this observation could be the high potency of CRT/E7, as we have previously demonstrated its robust biological efficacy in augmenting tumor-specific CD8+ responses [43]. This underscores the significance of HPV vaccination as a preventative measure against HPV-associated cancers.

Nevertheless, there are two primary limitations in this study to address. Firstly, our tumor experiments were conducted in hosts with inherent STAT1^−/−^, so the STAT1 expression in the tumor microenvironment remained unaltered. It is important to clarify this distinction because STAT1 expression impacts the host immune system and tumor microenvironment differently. Hosts with normal or activated STAT1 expression bolster immune surveillance, as discussed previously. However, activated STAT1 expression in the tumor microenvironment is positively correlated with the upregulation of PD-L1, thus suppressing intratumoral T-cell activities [58,59]. Our study strictly focuses on the effect of STAT1 expression on the host immune system, and not on the tumor microenvironment. Secondly, our tumor model is an HPV E6/E7-expressing sarcoma, representing only one type of HPV-associated cancers. Future efforts should aim to replicate this study in other types of E6/E7-expressing tumor models.

Previous studies have investigated the spontaneous development of mammary adenocarcinoma and lymphatic lesions in STAT1^−/−^ mice [56,60]. Our current study illustrates an understanding of the role of STAT1 on treatment responses via an HPV E6/E7-expressing spontaneous oral tumor model. Our findings demonstrate that spontaneous tumor growth was more rapid when using STAT1^−/−^ mice. Although the host immune response is weaker in STAT1^−/−^ mice, CRT/E7 vaccination is still sufficient to induce a controlled tumor response. In general, our findings suggest that immunocompetency is maintained against immunotherapeutics in STAT1^−/−^ hosts.

## Data Availability

Raw data were generated at Johns Hopkins University School of Medicine. The datasets used and/or analysed during the current study are available from the corresponding author on reasonable request.

## Supplementary Materials

The following supporting information can be downloaded at: https://www.mdpi.com/article/10.3390/vaccines12040430/s1, Figure S1: Representative flow gating and quantification of TIM3-specific CD4+ T cells for the indicated treatment groups.

## Abbreviation

CRT/E7	Calreticulin-E7
EGCG	Epigallocatechin 3-gallate
ICB	Immune checkpoint blockade
IFN	Interferon
IP	Intraperitoneal injection
JAK	Janus protein tyrosine kinase
HNSCC	Head and neck squamous cell carcinoma
HPV	Human papillomavirus
HR-HPV	High-risk human papillomavirus
MHC	Major histocompatibility complex
NK cells	Natural killer cells
NSCLC	Non-small-cell lung cancer
PBMC	Peripheral blood mononuclear cell
PD-1	Programmed cell death protein 1
PD-L1	Programmed death-ligand 1
RBC	Red blood cell
SH2 domain	Src homology 2 domain
STAT	Signal transducer and activator of transcription
TIL	Tumor-infiltrating lymphocyte
TNF-α	Tumor necrosis factor alpha
