# Supplementary figures and images for: STAT1-Deficient HPV E6/E7-Associated Cancers Maintain Host Immunocompetency against Therapeutic Intervention
Source: Vaccines (Basel). 2024 Apr 17;12(4):430. doi: 10.3390/vaccines12040430 (PMC11053987; doi:10.3390/vaccines12040430)

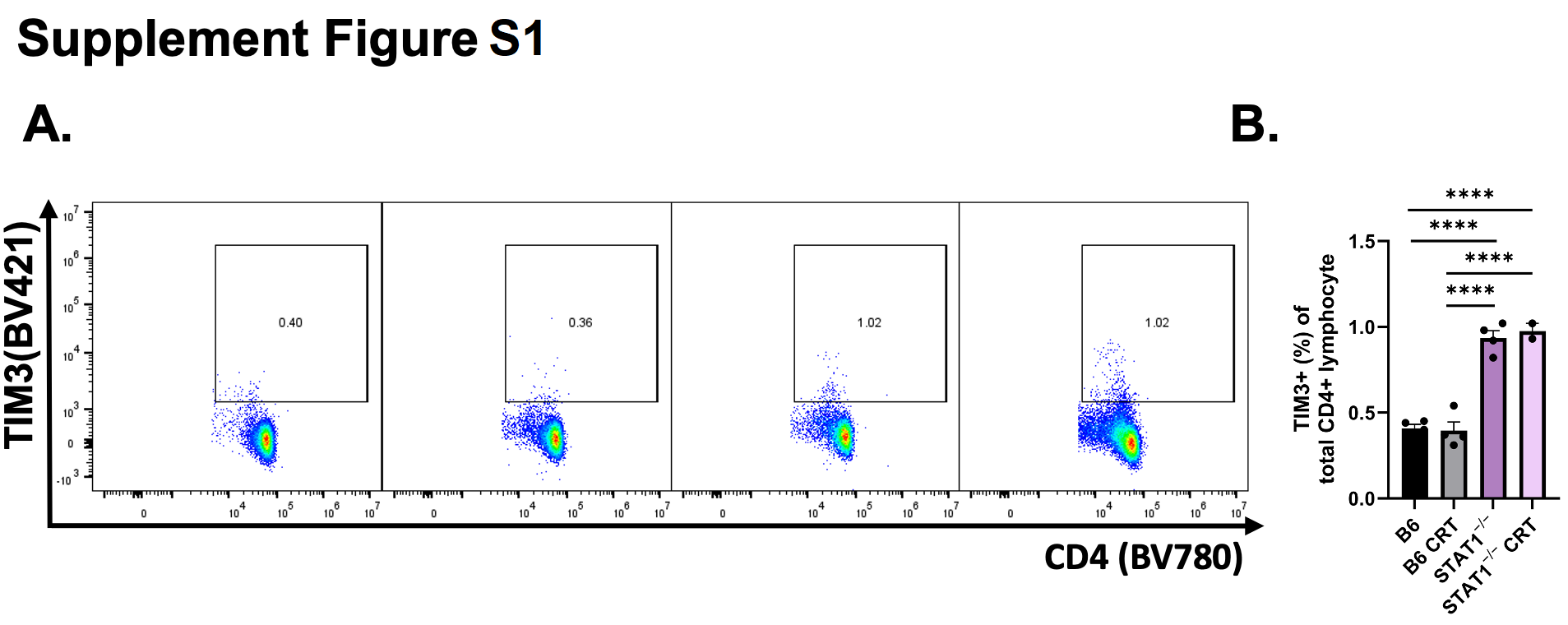

Supplement: Supplementary file 1 [file vaccines-12-00430-s001.zip › vaccines-2926614-supplementary.tiff]
